# Diabetes mellitus and the risk of vulvar and vaginal cancer: results from a large cohort study

**DOI:** 10.1186/s12905-026-04429-8

**Published:** 2026-03-28

**Authors:** Dagfinn Aune, Wei-Chuan Chang, Tatiana V. Macfarlane, Jakub G. Sobiecki, Tsung-Cheng Hsieh, Wen-Lin Hsu, Fang-Ling Chang, Ming-Shan He

**Affiliations:** 1 Department of Research, Cancer Registry of Norway, Norwegian Institute of Public Health, Oslo, Norway; 2 Department of Nutrition, Oslo New University College, Oslo, Norway; 3 Department of Epidemiology and Biostatistics, School of Public Health, Imperial College London, London, UK; 4 Department of Medical Research, Buddhist Tzu Chi General Hospital, Hualien, Taiwan; 5 Institute of Medical Sciences, Tzu Chi University, Hualien, Taiwan; 6 School of Medicine, Tzu Chi University, Hualien, Taiwan; 7 Department of Radiation Oncology, Buddhist Tzu Chi General Hospital, Hualien, Taiwan; 8 Department of Ophthalmology, Buddhist Tzu Chi General Hospital, No. 707, Sec. 3 Chung-Yung Road, Hualien, 970 Taiwan; 9 Department of Ophthalmology and Visual Science, Tzu Chi University, Hualien, Taiwan; 10 Department of Ophthalmology, Mennonite Christian Hospital, Hualien, Taiwan

**Keywords:** Diabetes mellitus, Vulvar cancer, Vaginal cancer, Cohort, Taiwan

## Abstract

**Background:**

Although diabetes mellitus has been associated with increased risk of several cancers, evidence in relation to vulvar and vaginal cancers has been limited and inconsistent. We investigated the association between diabetes and the risk of vulvar and vaginal cancer risk in a nationwide cohort of 2.8 million women in Taiwan.

**Methods:**

A retrospective cohort study of 2.8 million women with and without diabetes mellitus aged 18–90 years was conducted. Multivariable proportional hazards regression models were used to estimate hazard ratios (HRs) and 95% confidence intervals (CIs) for the association between diabetes diagnosis and the risk of vulvar and vaginal cancer.

**Results:**

During 7.8 years follow-up, 553 vulvar and 506 vaginal cancer cases occurred. A history of diabetes mellitus was associated with higher risk of vulvar cancer with a HR (95% CIs) of 1.34 (1.12–1.60), but not vaginal cancer (1.09, 0.91–1.31). The HR was 1.97 (0.95–4.11) for early-onset and 1.34 (1.12–1.61) for later-onset vulvar cancer and 1.81 (0.76–4.32) for early-onset and 1.09 (0.90–1.31) for later-onset vaginal cancer. The associations were similar when restricted to type 2 diabetes cases, and when excluding the first 5 years of follow-up.

**Conclusion:**

These findings suggest diabetes mellitus is associated with increased risk of vulvar, but not vaginal cancer. Further studies are needed to clarify these findings.

## Introduction

The incidence of vulvar and vaginal cancers is low with 0.9 and 0.4 cases per 100,000 person-years, respectively [1]. Globally, 45,000 and 18,000 vulvar and vaginal cancer cases occurred in 2020 [1]. Risk factors include infection with human papilloma virus (HPV) [2, 3], sexual habits [2–4], genital warts [2–7], vulvar lichen sclerosis [3], and conditions related to immunosuppression including systemic lupus erythematosus [8], HIV [3], and organ transplantation [3, 9, 10], diethylstilbestrol use [11], alcoholism [12] and smoking [2, 4, 13]. Some cohorts have suggested higher body mass index is associated with increased risk of both vulvar and vaginal cancer [14–17].

Although a history of diabetes mellitus is an established risk factor for six cancers, including colorectal, liver, gallbladder, pancreatic, breast, and endometrial cancers [18], data on other and less common cancers is emerging. Studies on diabetes mellitus and risk of vulvar cancer have been somewhat limited in number and have shown conflicting results [19–23]. One large US cohort study show no clear association [19], while three studies reported non-significant positive associations with relative risks (RRs) ranging from 1.17 to 1.53 [20, 21, 23], and one registry-based retrospective cohort study from Sweden found a significant 61% increase in risk [22]. We are only aware of two cohort studies on vaginal cancer [19, 22], and one reported a non-significant positive association with a RR of 1.36 (0.45–4.12) [19], while a second registry-based cohort reported a significant positive association with a RR of 1.79 (1.27–2.53) [22]. We are not aware of previous studies on diabetes and early-onset or later-onset vulvar or vaginal cancer risk. Given the rarity of these cancers further large scale cohort studies are therefore needed to clarify these associations. We therefore investigated the association between a history of diabetes mellitus and risk of vulvar and vaginal cancers, including early-onset and later-onset diagnosis, in a large cohort of 2.8 million Taiwanese women who were part of the Taiwan National Health Insurance Research Database.

## Methods

### Data sources

In this nationwide retrospective cohort study we used data from the Taiwan National Health Insurance Research Database (NHIRD) between January 2011 and December 2022 to analyse the association between diabetes mellitus and vulvar and vaginal cancer. The National Health Insurance program was launched on March 1, 1995 and by 2014, 99.9% of Taiwan’s population were covered by the registry, covering approximately 27.2 million individuals [24]. The NHIRD includes registration files and original claims data for reimbursements and collects data from almost all medical facilities in Taiwan, and the database was made available for public research projects from 2002. The database contains data on outpatient and inpatient claims, patient identification number, birth date, sex, treatment, dates of admission and discharge, date of death, and diagnostic codes according to the International Classification of Diseases, 9th revision, Clinical Modification (ICD-9-CM) codes before 2016 and ICD-10-CM since 2016. The data in the database was encrypted to protect the privacy of the individuals, and each patient has a unique encrypted identifier that was linked to the Taiwan Cancer Registry and National Death Registry to obtain information on cancer diagnoses and deaths. All datasets were interlinked using patient identification numbers.

### Study cohort

**Fig. 1:**
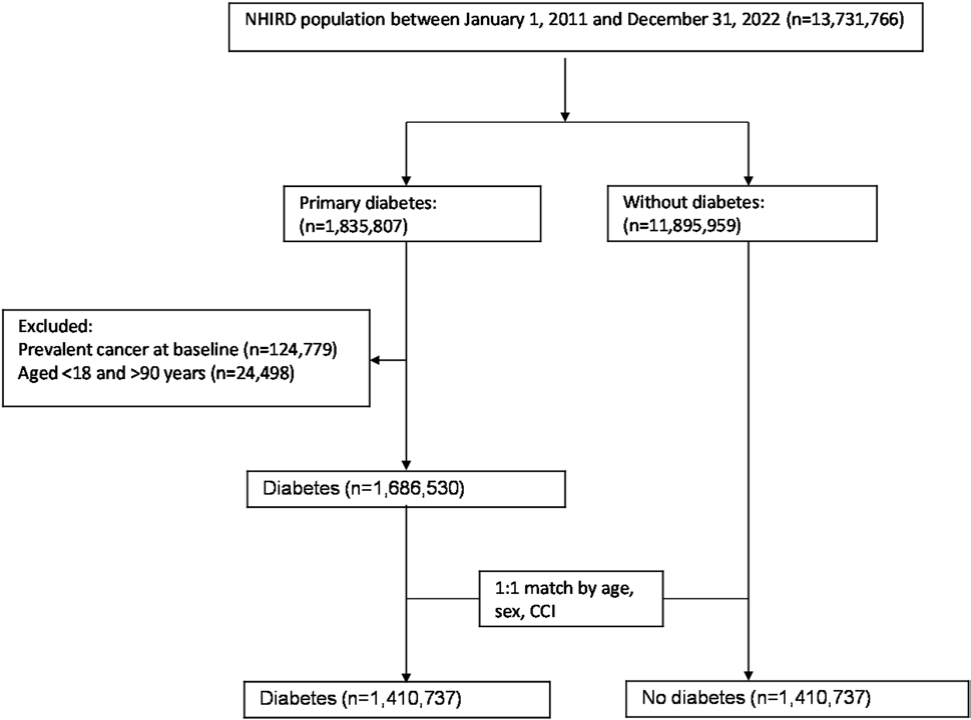
Flow-chart of participant selection and matching

This project was approved by the Institutional Review Board of Tzu Chi Hospital, Hualien (TCHIRB109-108-C). The requirement of informed consent was waived in accordance with the institutional guidelines. Among 13,731,766 women in the NHIRD between January 1, 2011 and December 31, 2022, 1,835,807 women with primary diabetes (ICD-9-CM 250 or ICD-10-CM E10, E11) and 11,895,959 women without diabetes were included. Participants with prevalent cancer at baseline and aged < 18 and > 90 years were excluded. Overall 1,686,530 women with primary diabetes remained after these exclusions. From the remaining participants, we selected randomly participants with and without diabetes matched in a 1:1 ratio by exact age, and Charlson comorbidity index [25], leaving 1,410,737 women with diabetes and 1,410,737 women without diabetes included in the analysis (Fig. 1).

### Outcome measures and follow-up

The outcome measure was incidence of vulvar or vaginal cancer, and cases were identified by linkage to the Taiwan Cancer Registry using International Statistical Classification of Diseases (ICD-10) codes C51 and C52, and ICD-9 codes 184.1-184.4 and 184.0, respectively. Only the first occurrence of these cancers was included. The participants were followed up from the index date, which was defined as the date of first recorded diabetes diagnosis for diabetes cases, and for non-diabetic participants the index date corresponded to that of the matched diabetes case, and the earliest possible entry was January 2011. The follow-up ended at the date of vulvar or vaginal cancer diagnosis, date of other cancer diagnosis, date of death or date of censoring (31 December 2022), whichever occurred first.

### Statistical analysis

Multivariable Cox proportional hazards regression models were used to estimate hazard ratios (HRs) and 95% confidence intervals (95% CIs) for the association between a history of diabetes mellitus vs. no diabetes mellitus and risk of vulvar and vaginal cancer, with time since the index date as the time scale. Participants with diabetes mellitus were matched with those without diabetes mellitus by age and Charlson Comorbidity Index, and the analyses were further adjusted for obesity, hypertension and hyperlipidemia. To take into account potential detection or surveillance bias, further adjustment was made for frequency of gynecological and urological outpatient visits before the cancer diagnosis in a second model. The proportional hazards assumption was tested for using Schoenfeld’s test and there was no violation (*p* = 0.30 and *p* = 0.19 for vulvar and vaginal cancer, respectively). We conducted additional analyses using only type 2 diabetes cases (there were too few cases to separately assess type 1 diabetes), for early-onset (< 50 years) vs. later-onset (≥ 50 years) cancer diagnosis, and stratified by duration of follow-up (< 1 year, 1-<5 years, ≥ 5 years). We used E-values to quantify the strength that an unadjusted confounder would need to have with both the exposure and the outcome to explain away the observed associations [26]. The statistical analyses were conducted using SAS version 9.4 for Windows (SAS Institute, Inc., Cary, NC, USA). All statistical tests were two-sided and a *p*-value < 0.05 was considered statistically significant.

## Results

A total of 2,821,474 women were included in the analysis of diabetes mellitus and risk of vulvar and vaginal cancer. Women with diabetes mellitus were more likely to have obesity, hypertension and hyperlipidemia than women without diabetes mellitus (Table 1). During 7.8 years follow-up, 553 vulvar and 506 vaginal cancer cases occurred.

**Table 1:** Baseline table

	Diabetes	
	No diabetes	Diabetes
Women, n (%)	1,410,737 (50)	1,410,737 (50)
Age, years, mean (SD)	59.46 (13.38)	59.46 (13.38)
Charlson Comorbidity Index, mean (SD)	0.02 (0.15)	0.02 (0.15)
Obesity, n (%)	510 (0.04)	4,685 (0.33)
Hypertension, n (%)	50,074 (3.55)	200,563 (14.22)
Hyperlipidemia, n (%)	15,955 (1.13)	79,891 (5.66)
Frequency of outpatient gynecological and urological visits prior to cancer diagnosis, mean (SD)	5.09 (12.90)	7.55 (16.42)

In the multivariable model 1, matched for age and Charlson Comorbidity Index and adjusted for obesity, hypertension and hyperlipidemia, a history of diabetes mellitus was positively associated with vulvar cancer with a HR (95% CIs) of 1.34 (1.12–1.60), but was not clearly associated with vaginal cancer risk with a HR (95% CI) of 1.09 (0.91–1.31) (Table 2). The E-value for the main analysis of diabetes and vulvar cancer was 2.01 (lower CI: 1.49). These associations persisted when only type 2 diabetes cases were analysed (HR, 95% CI: 1.34, 1.12–1.60 for vulvar cancer and 1.09, 0.91–1.31 for vaginal cancer, results not shown). When analyses were stratified by age of cancer diagnosis, there were non-statistically significant associations with early-onset vulvar (HR, 95% CI: 1.97, 0.95–4.11) and vaginal (1.81, 0.76–4.32) cancers, while the associations with later-onset cancers were similar to the main analysis (Table 3). These associations were similar when the first 5 years of follow-up were excluded (Table 4). Analyses with further adjustment for frequency of gynecological or urological visits prior to cancer diagnosis (model 2) only slightly modified these associations (Tables 2, 3 and 4), e.g. the main analysis showed a HR of 1.33 (1.11–1.58) for vulvar cancer and 1.07 (0.89–1.29) for vaginal cancer.

**Table 2:** Diabetes and vulvar and vaginal cancer risk

Outcome	Diabetes status	Cases	Participants	Person-years	Incidence rate per 100,000 person-years	HR (95% CI), model 1	HR (95% CI), model 2
Vulvar cancer	No diabetes	226	1,410,737	11,488,962	1.97	1.00 (ref.)	1.00 (ref.)
	Diabetes	327	1,410,737	10,942,989	2.99	1.34 (1.12–1.60)	1.33 (1.11–1.58)
Vaginal cancer	No diabetes	224	1,410,737	11,488,944	1.95	1.00 (ref.)	1.00 (ref.)
	Diabetes	282	1,410,737	10,943,099	2.58	1.09 (0.91–1.31)	1.07 (0.89–1.29)

Model 1: Analyses were matched for age, Charlson Comorbidity Index, and adjusted for obesity, hypertension and hyperlipidemia

Model 2: Model 1 + further adjustment for frequency of gynecological or urological visits prior to cancer diagnosis

**Table 3:** Diabetes and early-onset and later-onset vulvar and vaginal cancer risk

Outcome	Subgroup	Diabetes status	Cases	Person-years	Incidence rate per 100,000 person-years	HR (95% CI), model 1	HR (95% CI), model 2
Vulvar cancer	Early-onset	No diabetes	11	2,234,049	0.49	1.00 (ref.)	1.00 (ref.)
		Diabetes	26	2,215,606	1.13	1.97 (0.95–4.11)	2.04 (0.97–4.29)
	Later-onset	No diabetes	215	9,254,913	2.32	1.00 (ref.)	1.00 (ref.)
		Diabetes	302	8,727,383	3.46	1.34 (1.12–1.61)	1.32 (1.10–1.58)
Vaginal cancer	Early-onset	No diabetes	8	2,234,072	0.36	1.00 (ref.)	1.00 (ref.)
		Diabetes	18	2,215,630	0.81	1.81 (0.76–4.32)	1.74 (0.72–4.20)
	Later-onset	No diabetes	216	9,254,873	2.33	1.00 (ref.)	1.00 (ref.)
		Diabetes	264	8,727,469	3.02	1.09 (0.90–1.31)	1.07 (0.88–1.29)

Model 1: Analyses were matched for age, Charlson Comorbidity Index, and adjusted for obesity, hypertension and hyperlipidemia

Model 2: Model 1 + further adjustment for frequency of gynecological or urological visits prior to cancer diagnosis

**Table 4:** Diabetes and vulvar and vaginal cancer risk, analyses stratified by duration of follow-up

Outcome	Subgroup	Diabetes status	Cases	Person-years	Incidence rate per 100,000 person-years	HR (95% CI), model 1	HR (95% CI), model 2
Vulvar cancer	< 1 year	No diabetes	43	31,145	138.06	1.00 (ref.)	1.00 (ref.)
		Diabetes	49	36,078	135.82	0.82 (0.54–1.26)	0.75 (0.49–1.17)
	1-<5 years	No diabetes	80	904,682	8.84	1.00 (ref.)	1.00 (ref.)
		Diabetes	125	985,124	12.69	1.26 (0.94–1.68)	1.28 (0.96–1.72)
	≥ 5 years	No diabetes	103	10,553,135	0.98	1.00 (ref.)	1.00 (ref.)
		Diabetes	153	9,921,787	1.54	1.42 (1.10–1.84)	1.37 (1.05–1.78)
Vaginal cancer	< 1 year	No diabetes	44	31,139	130.28	1.00 (ref.)	1.00 (ref.)
		Diabetes	47	36,076	141.30	0.76 (0.50–1.17)	0.67 (0.43–1.04)
	1-<5 years	No diabetes	85	904,671	9.40	1.00 (ref.)	1.00 (ref.)
		Diabetes	115	985,109	11.67	1.04 (0.78–1.39)	1.06 (0.79–1.42)
	≥ 5 years	No diabetes	95	10,553,134	0.90	1.00 (ref.)	1.00 (ref.)
		Diabetes	120	9,921,914	1.21	1.06 (0.80–1.41)	1.01 (0.76–1.35)

Model 1: Analyses were matched for age, Charlson Comorbidity Index, and adjusted for obesity, hypertension and hyperlipidemia

Model 2: Model 1 + further adjustment for frequency of gynecological or urological visits prior to cancer diagnosis

## Discussion

We found a positive association between a history of diabetes mellitus and risk of vulvar cancer, but not with vaginal cancer in this large-scale cohort study of 2.8 million Taiwanese women. The associations were similar to the main analysis when restricted to type 2 diabetes cases, when assessing later-onset cancer diagnoses and when excluding the first 5 years of follow-up, while associations with early-onset cancers were imprecisely estimated.

Previous studies on diabetes mellitus and risk of vulvar and vaginal cancer have shown mixed results [19–23]. A large Swedish registry-based cohort found a 61% increase in risk of vulvar cancer among women with diabetes, while a few other studies reported non-significant positive associations [20, 21, 23], and one large US cohort found no association [19]. Similarly, a large Swedish registry-based cohort reported a 79% increase in risk of vaginal cancer [22], while a second study reported a non-significant positive association [19]. It is possible that several of these studies have had too limited statistical power to detect a clear association, and this may also be the case in the current study in spite of a relatively large sample size. Because of a lack of information on other risk factors such as smoking and human papilloma virus (HPV) infection, we cannot exclude the possibility that residual confounding may have influenced the results, and it is possible that the observed associations may have been overestimated given the positive associations observed between smoking and HPV infection and diabetes [27, 28] and vulvar cancer [3, 13]. In addition, although we adjusted for registry-based obesity status, this may be a crude indicator that may underestimate the true prevalence of obesity and residual confounding because of imprecise measurement is possible. The estimated E-value was 2.01 (lower CI: 1.49), suggesting that an unadjusted risk factor with a relatively strong association with both diabetes and vulvar cancer potentially could explain away the observed association. Interestingly, a recent study in Norway reported strong positive associations between higher body mass index and risk of early-onset vaginal cancer and with vulvar cancer overall, which is partly in line with the current findings on diabetes [15], considering the strong relation between higher body mass index and diabetes risk [29]. We did not adjust for vulvar lichen sclerosus, which is a strong risk factor for vulvar cancer [3], because there is some evidence that diabetes may increase risk of this condition [30], and it could therefore on the biological pathway from diabetes to vulvar cancer. Patients with diabetes are prone to genitourinary infections, which can lead to more frequent clinical follow-ups and pelvic examinations, which could result in detection or surveillance bias. We also tested whether detection or surveillance bias could explain the positive association between diabetes and vulvar cancer, however, further adjustment for frequency of gynecological and urological outpatient visits did not materially alter the observed associations. Strengths of the current study include the large sample size and linkages to a cancer registry with high-quality data that ensured the outcome assessment was complete.

Some of the mechanisms that have been hypothesized to explain a potential association between diabetes mellitus and vulvar and vaginal cancer include reduced immune function and ability to clear infections (e.g. HPV infection) [31, 32], increased risk of vulvar lichen sclerosus [30], which is a strong risk factor for vulvar cancer [3], and increased risk of genital warts in diabetes patients [33], which has been found to be strongly associated with increased risk of vulvar and vaginal cancer [6]. However, further studies are needed to investigate these associations as well as the underlying mechanisms. From a public health perspective, it could be argued that a hazard ratio of 1.34 for a relatively rare cancer may not be considered alarming by itself. Nevertheless, a history of diabetes is an established risk factor at least six other cancer types [18], and has established adverse impacts on a wide range of diseases and causes of death [34], thus adding up to a significant burden when assessing the evidence across a wider range of outcomes. In addition, adiposity is strongly associated with diabetes [29] and may be more strongly associated with vulvar and vaginal cancer [15] than diabetes itself, and if confirmed by further studies, the combination of adiposity and diabetes could be important targets in clinical practice or for screening strategies.

In summary, we found a positive association between a history of diabetes mellitus and the risk of vulvar cancer, but the association was null for vaginal cancer. Further studies are needed on diabetes and early-onset diagnosis of both cancers.

## Data Availability

Data are available from the National Health Insurance Research Database (NHIRD) published by the Taiwan National Health Insurance Bureau. Due to the legal restrictions imposed by the government of Taiwan in relation to the “Personal Information Protection Act”, data cannot be made publicly available. Requests for data can be sent as formal proposals to the NHIRD (http://nhird.nhri.org.tw).
